# Commensal Lactobacilli Enhance Sperm Qualitative Parameters in Dogs

**DOI:** 10.3389/fvets.2022.888023

**Published:** 2022-06-29

**Authors:** Feriel Yasmine Mahiddine, Inhwan You, Heekee Park, Min Jung Kim

**Affiliations:** Department of Research and Development, Mjbiogen Corp., Seoul, South Korea

**Keywords:** lactobacilli, sperm, canine, gut microbiome, probiotics

## Abstract

Although several methods have been developed to improve male fertility and sperm quality, subfertility remains a primary clinical issue in male reproduction worldwide. The aim of this study was to determine the effects of the oral administration of three commensal *Lactobacillus* spp. on healthy normozoospermic dogs and the qualitative parameters of their sperm. Three weeks of supplementation induced a significant decrease of two phyla, Proteobacteria and Tenericutes, and an increase of phylum Firmicutes. At the species level, the number of *Fusobacterium perfoetens* and *Anaerobiospirillum succiniciproducens* decreased, while *Limosilactobacillus reuteri* increased. Parallel to these results, qualitative sperm parameters such as total and progressive motility, acrosome integrity, and other kinematic parameters were significantly enhanced after commensal lactobacilli supplementation. In addition, we showed that Firmicutes were positively correlated with sperm qualitative parameters, while Proteobacteria, *F. perfoetens*, and *A. succiniciproducens* were negatively correlated. Considering the similarities between the gut microbiome of dogs and humans, these results provide more insight into how gut microbiota regulation could improve male sperm quality in both species.

## Introduction

Fertility is influenced by the health and lifestyle of an individual (1). Recent data show that male factor subfertility is responsible for fertility problems in 30% of cases (2). External factors, such as diet (3, 4) and stress (5), as well as internal factors such as aging (6), metabolism (7), and nutrient availability (8, 9), significantly influence reproductive function. These factors could affect the gut microbiome as well as spermatogenesis in males (10), which results in changes in sperm qualitative and quantitative parameters such as sperm morphology, viability, motility, and chromatin integrity. Accordingly, it was hypothesized that the management of gut microbiota could help increase sperm qualitative parameters, and subsequently, a link between the gut microbiome and testicular dysfunction through polyamine metabolism has been established (11). A recent study showed that triptolide-induced testicular dysfunction was successfully reversed by spermine supplementation or transplantation of bacterial strains that enhance spermine production in the gastrointestinal tract (11).

Canine male reproductive function is similar to that of human males, which makes dogs a good model for fertility issues, especially when considering the similarities in diseases, environment, life span, size, genetics, and anatomy (12, 13). Some of the genes involved in infertility and spermatogenesis, are found in both humans and dogs (14), and the morphological and qualitative abnormalities of sperm are similar in both species (15, 16). Further, the gut microbiome of both species is functionally and structurally similar (17, 18). Since their domestication, dogs have been sharing the same food sources as humans (19), and have switched their diet from carnivorous to omnivorous, which gradually modified their digestive and metabolic characteristics, making them closer to those of omnivorous mammals (20). This dietary change further influenced the microbiome of domestic dogs, making it more similar to that of humans. Dogs and humans gut microbiome have similar taxonomic profiles and distributions at the phylum and genus levels (17, 18, 21). The strong similarities between the two species suggest that studying the dog microbiome would help to uncover its roles not just in dogs but also in humans.

Lactobacilli are one of the beneficial commensal bacterial groups that are the most common type of probiotic organisms. They contribute to the maintenance of gut homeostasis by maintaining a beneficial microbial balance. These bacteria defend against colonization by opportunistic pathogens through the production of antimicrobial compounds and their high antioxidant ability (22). As oxidative stress is one of the main causes of subfertility in both dogs and humans (23–25), we hypothesized that commensal *Lactobacillus* spp. could enhance sperm quality in dogs. Although the effects of many dietary supplements on dog sperm have been evaluated before (26–28), probiotics effects have not been evaluated yet. We selected three commensal *Lactobacillus* spp., namely *Lactobacillus acidophilus, Limosilactobacillus reuteri* (previously known as *Lactobacillus reuteri*), and *Ligilactobacillus salivarius* (*Lactobacillus salivarius*), based on our previous study (21). All three species are currently used as probiotics and have been approved as feed additives in South Korea.

## Materials and Methods

### Dogs Selection

All the dogs used in this study belonged to the same owner. All experimental procedures in this study were performed with the consent of the owner. Nine poodles kept in the same environment were used in this study. They were fed commercial adult dry food once a day, and water was provided *ad libitum*. The dogs were selected based on different criteria including body condition scores of 4–5 on a 9-point scale, ages ranging from 2 to 9 years, health state, fertility records, and dogs with fertility issues were excluded from the study. All the dogs were healthy, vaccinated, de-wormed, free of diseases, and had no history of medication, diarrhea, or other medical conditions for 4 months prior to the start of the experiment.

### Bacteria Culture and Preparation of Supplementation

Three selected *Lactobacillus* spp., *L. acidophilus* KACC 12419, *L. salivarius* KACC 10006, and *L. reuteri* KACC 11452, were obtained from the Korean Agricultural Culture Collection (KACC). All the strains were identified via 16S rRNA sequencing prior to the experiments. Bacterial strains were cultured in De Man, Rogosa, and Sharpe (MRS) broth overnight at 37°C and washed twice using phosphate buffered saline. Then, bacterial pellets were resuspended in water and 20% glycerol and stored at −20°C in aliquoted tubes. Each tube contained the following concentrations: 3.0 × 10^9^ colony forming unit (CFU) of *L. acidophilus*, 2.7 × 10^8^ CFU of *L. salivarius*, and 1.3 × 10^9^ CFU of *L. reuteri*. The tubes were thawed at room temperature before use. All dogs were orally administered a mixture of the three commensal *Lactobacillus* spp., with fructo- and galactooligosaccharide (0.8% of food intake) along with dry food once a day, every day, for 3 weeks.

### Sample Collection and Processing

Rectal swab samples were collected at weeks 0 and 3 on the same days as sperm collection, using N-Swab transport (Noble Bio, Hwaseong, Korea). They were then transported to the laboratory at 4°C within 2 h. All the samples were stored at −80°C until further experiments. Microbial genomic DNA was extracted DNeasy PowerSoil Kit (Qiagen, Hilden, Germany). A bitch in estrus was used to stimulate the dogs, and semen samples were collected manually from each dog twice: before the start of dietary supplementation and 3 weeks after the start of the experiment (29). The first fraction was discarded, and the second fraction only, which represents the sperm-rich fraction, was collected and used for analysis. The samples were diluted with Tris-extender 1:1 (v/v) (distilled water, tris (hydroxymethyl) aminomethane 24 g/L, citric acid 14 g/L, fructose 8 g/L, and kanamycin sulfate 0.15 g/L; pH 6.6, 290 mOsm) and centrifuged at 700 × g for 1 min. Supernatants were collected and centrifuged (500 × g/5 min), and the pellets were resuspended in Tris-extender and chilling media [54% (v/v) Tris-extender, 40% (v/v) egg yolk, and 6% (v/v) glycerol]. The samples were chilled for 4–6 h at 4°C before being transported to the research facility (Mjbiogen) and processed for analysis (30).

### Gut Microbiota Profiling

Library construction, sequencing, and gut microbiome analysis were performed as previously described (21). Briefly, the Illumina 16S Metagenomic Sequencing Library Prep Guide was used for the V3-V4 region. Paired-end sequencing was performed by Macrogen (Seoul, South Korea) using the MiSeq™ platform (Illumina, San Diego, CA, USA). The sequences were trimmed and clustered into operational taxonomic units (OTUs) with 97% identity similarity, and microbial community analysis was performed using Quantitative Insights Into Microbial Ecology (QIIME) 1.9 (31).

### Sperm Kinematic Parameters Evaluation

Sperm kinematic parameters were evaluated using a computer-assisted sperm analysis system (Sperm Class Analyzer^®^ System version 6.4.0.93, Microptic, Barcelona, Spain), which included a Nikon Eclipse ci-L microscope (Nikon, Tokyo, Japan) with a 10 × phase-contrast objective, a heating stage at 37°C, with a frame rate set at 25 frames/s. Settings were adjusted according to the manufacturers recommendations (32), and a minimum of 500 cells in 5 random fields per sample were analyzed. For analysis, a sperm drop of 3 μl per sample was placed on Leja 20 μm chamber slides (Leja, Gynotec Malden, Nieuw Vennep, Netherlands) at 37° (33). Total sperm motility, progressive motility, curvilinear velocity (VCL), straight-line velocity (VSL), average path velocity (VAP), linearity (LIN), straightness (STR), wobble VAP/VCL (WOB), amplitude of lateral head (ALH), and beat cross frequency (BCF) were analyzed.

### Acrosome Integrity Assessment

Fluorescein isothiocyanate-conjugated peanut agglutinin (FITC-PNA) was used to assess the acrosome integrity, as described previously (34). Semen smears were fixed in absolute methanol, stained, and mounted with anti-fade mounting medium (VECTASHIELD^®^, Vector Laboratories, Burlingame, CA, USA). The integrity of the sperm acrosome membrane was evaluated in at least 100 cells per slide, using an epifluorescence phase-contrast microscope (Eclipse Ts 2, Nikon, Tokyo, Japan) and classified as intact (strong green fluorescence) or non-intact (partial or no fluorescence).

### Eosin-Nigrosin Staining

Eosin–nigrosin staining was performed to determine the proportion of live sperm cells and morphology defects. Briefly, samples were washed, and equal amounts of semen, eosin, and nigrosin were mixed and smeared onto warm glass slides. The slides were then air-dried, and 200 sperms were examined under a light microscope (Eclipse Ts 2, Nikon, Tokyo, Japan) with an oil immersion objective lens (1,000 × magnification). The unstained sperms were counted as alive, stained ones were counted as dead, and the results were expressed as the proportion of live sperm cells (35). Different morphological defects were counted in every slide, according to previous studies (15, 36). Briefly, sperm with a coiling of the mid piece were counted as cells with a coiled tail, the ones with a bending of the mid piece or the entire tail were counted as cells with a bent tail, and sperm with a droplet in the tail were counted as cells with a droplet. Cells with a head defect were counted as well.

### Statistical Analysis

Sperm parameters were statistically analyzed using GraphPad Prism 5.0 (GraphPad, CA, San Diego, USA). Prior to the analysis, D'Agostino and Pearson omnibus test was performed. The sperm data were analyzed using a one-tailed paired *t*-test. Alpha- and beta diversity were calculated based on observed OTUs and weighted UniFrac distances, respectively. Other analyses were performed using R software version 3.0.1. The Kruskal–Wallis test or Wilcoxon rank-sum test was used for bacterial relative abundance comparison. Pearson's correlation coefficient was calculated using *cor* function in the R software to measure the association between sperm qualitative parameters and gut microbiome abundance. Plotting was performed using the *Corrplot* function. All values are expressed as mean ± standard error of the mean (SEM), and values of *p* < 0.05 were considered statistically significant.

## Results

### Sperm Kinematic Parameters

Sperm kinematic parameters were assessed before the start of the experiment (week 0); after 3 weeks of supplementation (week 3), there was a significant improvement in sperm qualitative parameters (Table 1; *p* < 0.05). Total and progressive motility were the main qualitative parameters used to evaluate male fertility, and both were significantly enhanced at week 3 compared to week 0 (93.6 ± 3.6% vs. 90.5 ± 2.9% for total motility, and 60.4 ± 7.4% vs. 44.9 ± 7.5% for progressive motility). Other sperm kinematic parameters, such as VCL, VAP, VSL, LIN, STR, and BCF, were also significantly enhanced (Table 1).

**Table 1:** Sperm qualitative parameters in the first and last week of supplementation.

**Parameters**	**Week 0**	**Week 3**	***p*-value**	**Significance**
Motility (%)	90.5 ± 2.9	93.6 ± 3.6	0.02	*
Progressive motility (%)	44.9 ± 7.5	60.4 ± 7.4	0.00	*
VCL (μm/s)^a^	95.0 ± 10.1	110.3 ± 11.9	0.03	*
VAP (μm/s)	52.6 ± 5.1	61.1 ± 6.1	0.01	*
VSL (μm/s)	32.2 ± 3.4	40.8 ± 4.1	0.01	*
LIN (%)	33.1 ± 1.6	35.9 ± 2.1	0.04	*
STR (%)	57.1 ± 2.1	62.3 ± 2.2	0.01	*
WOB (%)	56.4 ± 1.0	55.6 ± 1.3	0.79	NS
ALH (μm)	2.5 ± 0.2	2.8 ± 0.3	0.06	NS
BCF (Hz)	10.0 ± 0.8	11.5 ± 1.0	0.03	*
Live cells (%)	65.4 ± 5.6	73.0 ± 3.8	0.1	NS
Intact acrosome (%)	83.8 ± 3.3	92.9 ± 1.9	0.03	*

*^a^VCL, average curvilinear velocity; VSL, straight-line velocity; VAP, average path velocity; LIN, linearity (average ratio of VSL/VCL); STR, straightness (average value of the ratio VSL/VAP); WOB, wobble; ALH, amplitude of lateral head; BCF, beat cross frequency.*

All results show means ± SEM. Values with a “*” are significantly different (p <0.05, n = 9), while values with “NS” are non-significant.

### Viability, Acrosome Integrity, and Morphological Defects

A significant increase in acrosome integrity was seen after commensal lactobacillus supplementation (84 ± 3.4% at week 0 vs. 92.9 ± 1.9% at week 3) (*p* < 0.05), although there was no difference in cell viability (65.7 ± 5.6% at week 0 vs. 73 ± 3.8% at week 3) (Table 1). As for the morphological defects, coiled and bent tails proportions were significantly reduced at week 3 (5.6 ± 0.9% and 12.3 ± 5.0% at week 0, 3.0 ± 0.4% and 3.3 ± 1.0% at week 3 respectively). Tail droplets were also reduced at week 3 (2.6 ± 1.2% vs. 0.2 ± 0.0%), but there was no significant reduction in head defects proportion (0.4 ± 0.3% vs. 0.0 ± 0.0%) (Table 2).

**Table 2:** Sperm morphological defects in the first and last week of supplementation.

**Morphological defects**	**Week 0**	**Week 3**
Head (%)	0.4 ± 0.3	0.0 ± 0.0
Droplets (%)	2.6 ± 1.2^a^	0.2 ± 0.0^b^
Coiled tail (%)	5.6 ± 0.9^a^	3.0 ± 0.4^b^
Bent tail (%)	12.3 ± 5.0^a^	3.3 ± 1.0^b^

*All results show means ± SEM. Values within marked with the letters “a” or “b” are significantly different (p <0.05, n = 9)*.

### Gut Microbiota Diversity and Composition

**Figure 1:**
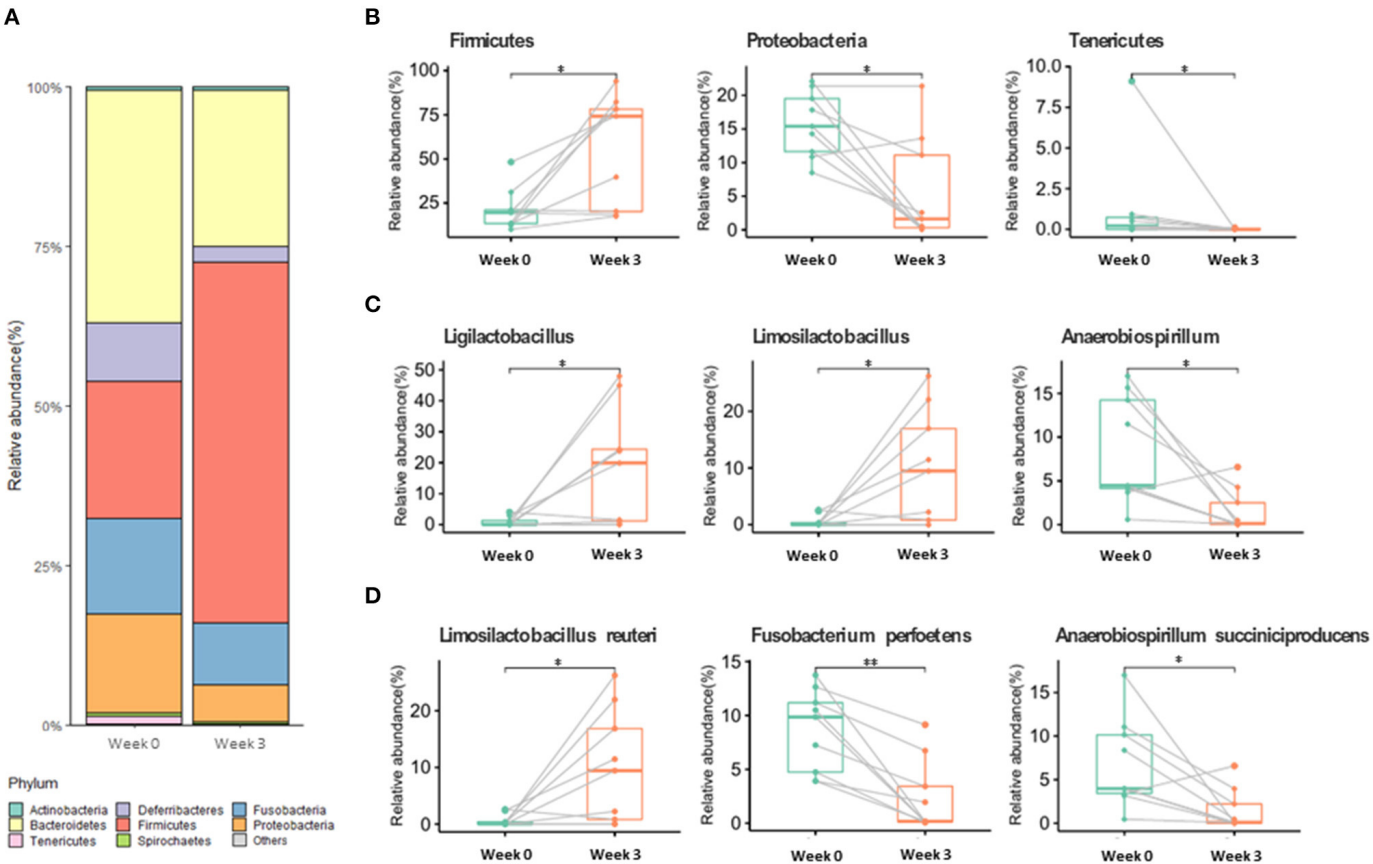
Gut microbiome relative abundance at the phylum **(A,B)**, genus **(C)**, and species **(D)** levels in week 0 and week 3. Values with “*” (*p* < 0.05) or “**” (*p* < 0.01) are significantly different.

Among the alpha diversity parameters, the Shannon index was statistically different before and after supplementation (Supplementary Figure 2). For beta diversity, weeks 0 and 3 tended to be clustered (Supplementary Figure 2). Gut microbiota composition at week 3 significantly changed in comparison with that at week 0 (Figure 1; Supplementary Table 1) (*p* < 0.05). At the phylum level, the relative abundance of Firmicutes significantly increased at week 3 compared to that at week 0, whereas Proteobacteria and Tenericutes significantly decreased at week 3 compared to that at week 0. At the genus level, *Ligilactobacillus* and *Limosilactobacillus* were significantly enhanced after supplementation (Figure 1), whereas *Anaerobiospirillum* was significantly decreased. At the species level, *Limosilactobacillus reuteri* was significantly enhanced at week 3, whereas *Fusobacterium perfoetens* and *Anaerobiospirillum succiniciproducens* were significantly decreased (Figure 1).

### Correlation Between Gut Microbiota and Sperm Parameters

**Figure 2:**
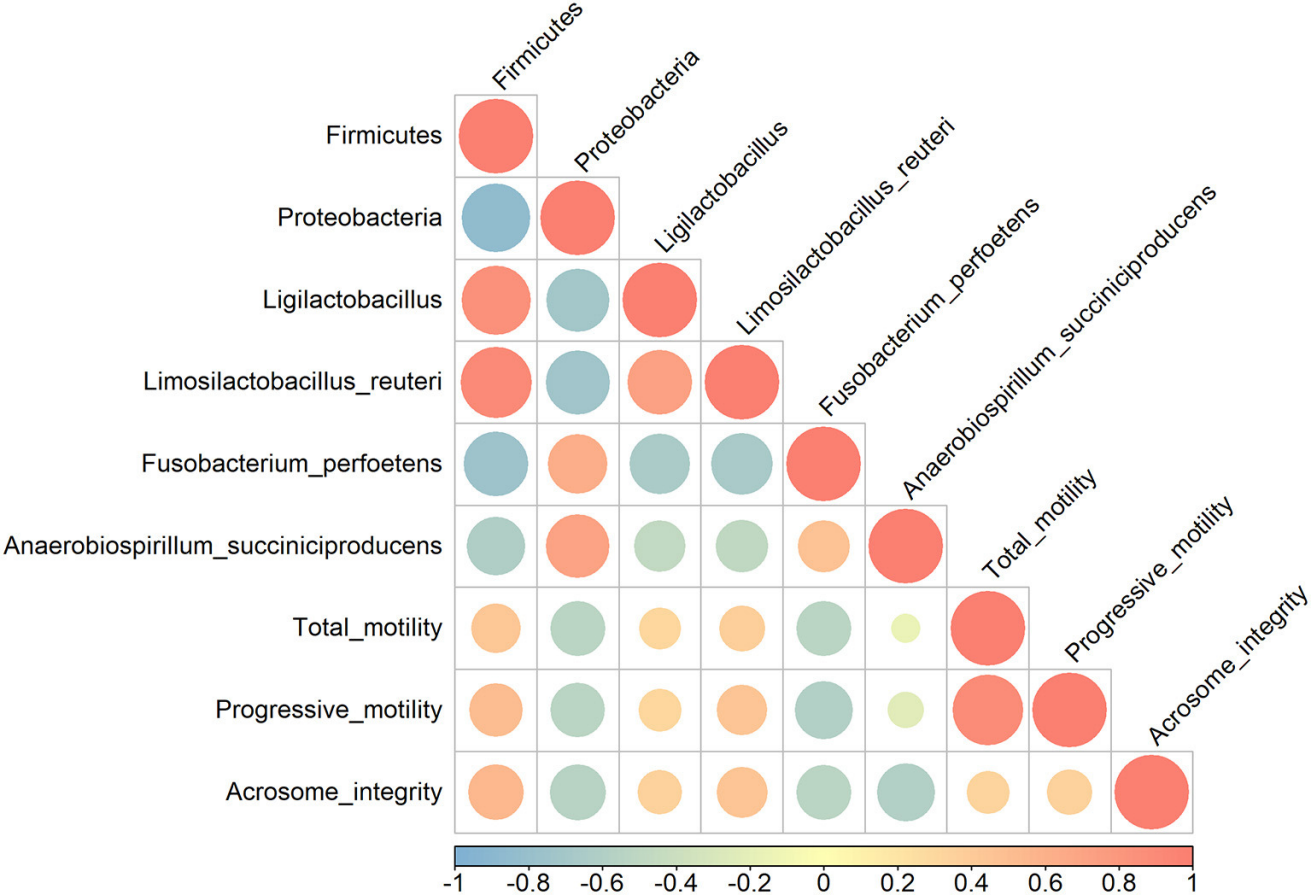
Pearson correlation analysis between relative abundance of bacterial phyla and species, and sperm qualitative parameters. Coefficients are colored from light blue (negative correlation) to light red (positive correlation), and color intensity and circle sizes are proportional to the level of correlation.

Pearson's correlation coefficients were calculated to evaluate possible correlations between sperm qualitative parameters and gut microbiome populations that were significantly altered (Figure 2). Firmicutes had a moderate positive correlation with sperm progressive motility and acrosome integrity (*r* = 0.50 and *r* = 0.54, respectively) (*p* < 0.05), whereas Proteobacteria showed a moderate negative correlation with sperm motility, progressive motility, and acrosome integrity (*r* = −0.53, *r* = −0.53 and *r* = −0.56) (*p* < 0.05). Qualitative sperm parameters showed a moderate negative correlation with *F. perfoetens* (*r* = −0.54, *r* = −0.59 and *r* = −0.54) (*p* < 0.05). A weak negative correlation was observed between *A. succiniciproducens* and acrosome integrity (*r* = −0.60) (*p* < 0.05). Other results were not significantly different.

## Discussion

Probiotics are used as dietary supplements to improve and regulate the gastrointestinal microbiome by promoting the growth of beneficial bacteria, inhibiting the growth of pathogens and potentially harmful microorganisms (37). Gut commensal lactobacilli such as *L. acidophilus, L. reuteri, L. rhamnosus, L. plantarum, and L. salivariu*s have been used as probiotic supplements in recent years and have a positive effect on the general health and gut microbiome health of the host (38–42). Among the various *Lactobacillus* spp., we selected three of them (*L. acidophilus, L. reuteri*, and *L. salivarius*) as commensal probiotics based on our previous study (21), and a list of approved feed additives in South Korea. Dogs were fed with commensal lactobacilli for 3 weeks as a preliminary study, to evaluate the effects of probiotics after only one cycle of spermatogenesis. A previous study showed that probiotics supplementation significantly improved sperm quality in zebrafish after one spermatogenetic cycle (43). Considering that one canine spermatogenic cycle duration is of approximately 2 weeks (44), and that 4–4.5 cycles are necessary to complete the entire spermatogenesis process in dogs, the observed changes indicate that probiotics supplementation starts to affect sperm quality after one spermatogenesis cycle. Similarly, supplementation of omega 3 and vitamin E significantly improved canine sperm quality after 2 weeks of supplementation (26). Our results could be due to an improvement in epididymal and prostatic fluid, and more research are needed to confirm this.

In the present study, changes in the gut microbiome and sperm qualitative parameters, as well as their correlation with commensal lactobacillus supplementation, were analyzed in normozoospermic dogs. We showed that a combination of three *Lactobacillus* spp., *L. acidophilus, L. reuteri, and L. salivarius*, could improve sperm kinematic parameters, including total and progressive motility (Table 1). The enhancement of these parameters might be due to the antioxidant abilities of lactobacillus. It has been reported that *L. salivarius* has antioxidant effects on sperm *in vitro* (45). However, the mechanisms by which it might affect sperm parameters *in vivo* have not yet been reported, and further research is needed to uncover them. The presence of an intact acrosome is required for a successful fertilization, as zona pellucida penetration is only possible after the release of enzymes from the acrosome (46). Although the importance of acrosome integrity cannot be ignored, there are no reports on the effects of lactobacillus supplementation on acrosomes. Here, we report an improvement in acrosome integrity after commensal lactobacillus supplementation (Table 1).

Sperm tail defects were significantly reduced after the 3rd week (Table 2). Although DNA integrity was not assessed in this study, a relationship between DNA fragmentation levels and tail abnormalities has been shown previously (47), which suggests that lactobacillus might have effects on DNA integrity as well. During this experiment, one dog was diagnosed with idiopathic infertility, and 3 weeks of commensal lactobacillus supplementation did not help restore fertility (data not shown). However, a previous study showed that prolonged supplementation with probiotics could restore fertility in infertile human patients; the administration of a combination of *L. acidophilus, L. bulgaricus, L. rhamnosus, L. casei, Bifidobacterium breve, B. longum, and Streptococcus thermophilus* significantly enhanced sperm motility, DNA integrity, and chromatin status in infertile men after 80 days of supplementation (48). Dogs were supplemented with commensal lactobacilli for 3 weeks only, to cover one spermatogenic cycle, while the total duration of the spermatogenesis process in dogs is estimated to last 8–9 weeks, covering 4–4.5 cycles (49). This could explain the absence of effects of probiotics supplementation on the infertile dog and point at the need of longer supplementation periods. Although sperm quality assessment is a fast and accessible procedure in small animal reproduction, it is not a valuable indicator of the fertility state, since fertility is a multifactorial parameter. Therefore, further experiments are required to assess the effects of probiotics on other factors.

Parallel to sperm analysis, gut microbiota composition and changes were also assessed to determine a possible link between changes in sperm qualitative parameters and gut health. Relative abundance changes at the phylum, genus, and species levels are examined in the Results section (Figure 1). The numbers of Proteobacteria and Tenericutes significantly decreased after 3 weeks of supplementation, while Firmicutes significantly increased. In other studies, Proteobacteria were linked with diseases and gut dysbiosis (50–52), and our findings further revealed that Proteobacteria are negatively correlated with sperm qualitative parameters (Figure 2). These results indicate a possible link between gut dysbiosis and the involvement of Proteobacteria in sperm quality. Additionally, Tenericutes are speculated to be involved in this process, although further confirmatory evidence is needed. Firmicutes contain an important polyamine called spermine (53). Spermine has a role in anti-oxidation and reproductive physiology homeostasis in males (53); it regulates the factors that protect against testicular injury, and oral supplementation with spermine successfully reversed testicular injury (11), which shows a direct link between gut microbiota and testicular tissue repair. In our results, there was a moderate positive correlation between Firmicutes and progressive motility and acrosome integrity. These elements suggest that commensal lactobacilli could be an interesting supplement to patients suffering from testicular trauma, to ensure a fast recovery of testicular function through the involvement of Firmicutes and spermine.

One of the more abundant species, *L. reuteri*, which was also included in our supplementation mix, may have easily settled in the intestine due to its commensal characteristics (Figure 1). *L. reuteri* has been reported to be associated with improved gut health (39, 40) and a reduction in the Proteobacteria population (40, 54), which is in accordance with our results (Figure 1). Moreover, it efficiently decreased intestinal inflammation (39, 40, 55), and improved digestive comfort (54, 56). However, to this date, there have been no reports on the role of *L. reuteri* in sperm qualitative parameters, and our results show no significant correlation between *L. reuteri* and sperm quality (Figure 2). *F. perfoetens* relative abundance decreased after commensal lactobacillus supplementation (Figure 1) and showed a strong negative correlation with total motility, progressive motility, and acrosome integrity (Figure 2). Considering that *F. perfoetens* is found in aged dogs (21), it can be suggested that commensal lactobacillus supplementation counteracts the effects of aging on reproductive function by regulating the relative abundance of *F. perfoetens*.

In conclusion, supplementation with commensal lactobacilli composed of *L*. *acidophilus, L*. *reuteri*, and *L*. *salivarius* in dogs successfully enhanced the viability of sperm kinematic, acrosome integrity, and modified gut microbiota populations, without clinical side effects such as vomiting or diarrhea. Overall, these findings provide insights into the influence of beneficial commensal lactobacillus supplementation on dog sperm quality parameters. Furthermore, some bacterial groups appear to be potential biomarker candidates for sperm quality. Based on the correlation results between gut microorganisms and sperm qualitative parameters, Firmicutes could be used as a positive marker for sperm quality, whereas Proteobacteria and *F. perfoetens* could be used as negative markers. Although this is a preliminary study with a small sample size and a short supplementation period, these results open up new research possibilities in the field of veterinary medicine, and further experiments are warranted to identify other bacterial populations and pathways that influence male reproductive function.

## Data Availability Statement

Supplementary data is available in the additional files and further supporting data is available from the authors on request.

## Ethics Statement

Ethical review and approval were not required for this study as noninvasive experiments were performed with the consent of the animal owners. Written informed consent was obtained from the owners for the participation of their animals in this study.

## Author Contributions

MK, FM, and IY: conceptualization, data curation, investigation, methodology, visualization, and writing—review and editing. MK and FM: formal analysis and validation. MK: supervision. MK and HP: funding acquisition, project administration, and resources. FM: writing—original draft. All authors contributed to the article and approved the submitted version.

## Funding

This study was supported by a Cooperative Research Program of Rural Development Administration (#PJ014786012022).

## Conflict of Interest

FM, IY, HP, and MK were employed by Mjbiogen Corp.

## Publisher's Note

All claims expressed in this article are solely those of the authors and do not necessarily represent those of their affiliated organizations, or those of the publisher, the editors and the reviewers. Any product that may be evaluated in this article, or claim that may be made by its manufacturer, is not guaranteed or endorsed by the publisher.
