# Supplementary material for: Commensal Lactobacilli Enhance Sperm Qualitative Parameters in Dogs
Source: Front Vet Sci. 2022 Jun 29;9:888023. doi: 10.3389/fvets.2022.888023 (PMC9278085; doi:10.3389/fvets.2022.888023)
Supplement: Supplementary file 1 [file Data_Sheet_1.docx]

Supplementary Material

# Supplementary Data

**Supplementary Table 1**. submission Relative abundance at the phylum, genus and species. Kruskal–Wallis test was used, and data are shown as the mean ± SEM.

| Bacteria | Week 0 | Week 3 | *p* value | FDR |
| --- | --- | --- | --- | --- |
| **Phylum** |  |  |  |  |
| Tenericutes | 1.34 ± 1.04 | 0.03 ± 0.01 | 0.01 | 0.05 |
| Firmicutes | 21.39 ± 4.07 | 56.58 ± 10.65 | 0.02 | 0.05 |
| Proteobacteria | 15.63 ± 1.68 | 5.83 ± 2.64 | 0.02 | 0.05 |
| **Genus** |  |  |  |  |
| *Bacteroides* | 7.95 ± 2.4 | 2.35 ± 1.21 | 0.004 | 0.06 |
| *Suttrella* | 2.84 ± 0.43 | 0.91 ± 0.35 | 0.004 | 0.06 |
| *Anaerobiospirillum* | 8.11 ± 2.07 | 1.36 ± 0.68 | 0.01 | 0.10 |
| *Anaeroplasma* | 1.34 ± 1.04 | 0.03 ± 0.01 | 0.01 | 0.10 |
| *Kineothrix* | 0.63 ± 0.14 | 0.29 ± 0.11 | 0.02 | 0.11 |
| *Enterococcus* | 0.00 ± 0.00 | 0.54 ± 0.32 | 0.02 | 0.11 |
| *Limosilactobacillus* | 0.61 ± 0.36 | 10.49 ± 3.51 | 0.03 | 0.14 |
| *Ligilactobacillus* | 0.98 ± 0.49 | 18.24 ± 6.36 | 0.04 | 0.14 |
| **Species** |  |  |  |  |
| *Fusobacterium perfoetens* | 8.61 ± 1.27 | 2.25 ± 1.01 | 0.004 | 0.07 |
| *Sutterella stercoricanis* | 2.84 ± 0.43 | 0.91 ± 0.35 | 0.004 | 0.07 |
| *Anaerobiospirillum thomasii* | 1.44 ± 0.73 | 0.08 ± 0.05 | 0.01 | 0.07 |
| *Bacteroides faecis* | 3.35 ± 0.75 | 1.28 ± 0.61 | 0.01 | 0.07 |
| *Anaerobiospirillum succiniciproducens* | 6.66 ± 1.75 | 1.27 ± 0.65 | 0.01 | 0.08 |
| *Anaeroplasma bactoclasticum* | 1.34 ± 1.04 | 0.03 ± 0.01 | 0.01 | 0.08 |
| *Bacteroides uniformis* | 3.34 ± 1.64 | 0.28 ± 0.23 | 0.02 | 0.08 |
| *Kineothrix alysoides* | 0.63 ± 0.14 | 0.29 ± 0.11 | 0.02 | 0.08 |
| *Enterococcus hirae* | 0.00 ± 0.00 | 0.53 ± 0.32 | 0.02 | 0.08 |
| *Limosilactobacillus reuteri* | 0.61 ± 0.36 | 10.35 ± 3.46 | 0.03 | 0.11 |
| *Ligilactobacillus apodemi* | 0.97 ± 0.49 | 18.16 ± 6.33 | 0.04 | 0.11 |
| *Phocaeicola plebeius* | 1.38 ± 0.38 | 0.57 ± 0.29 | 0.04 | 0.11 |

## Supplementary Figures


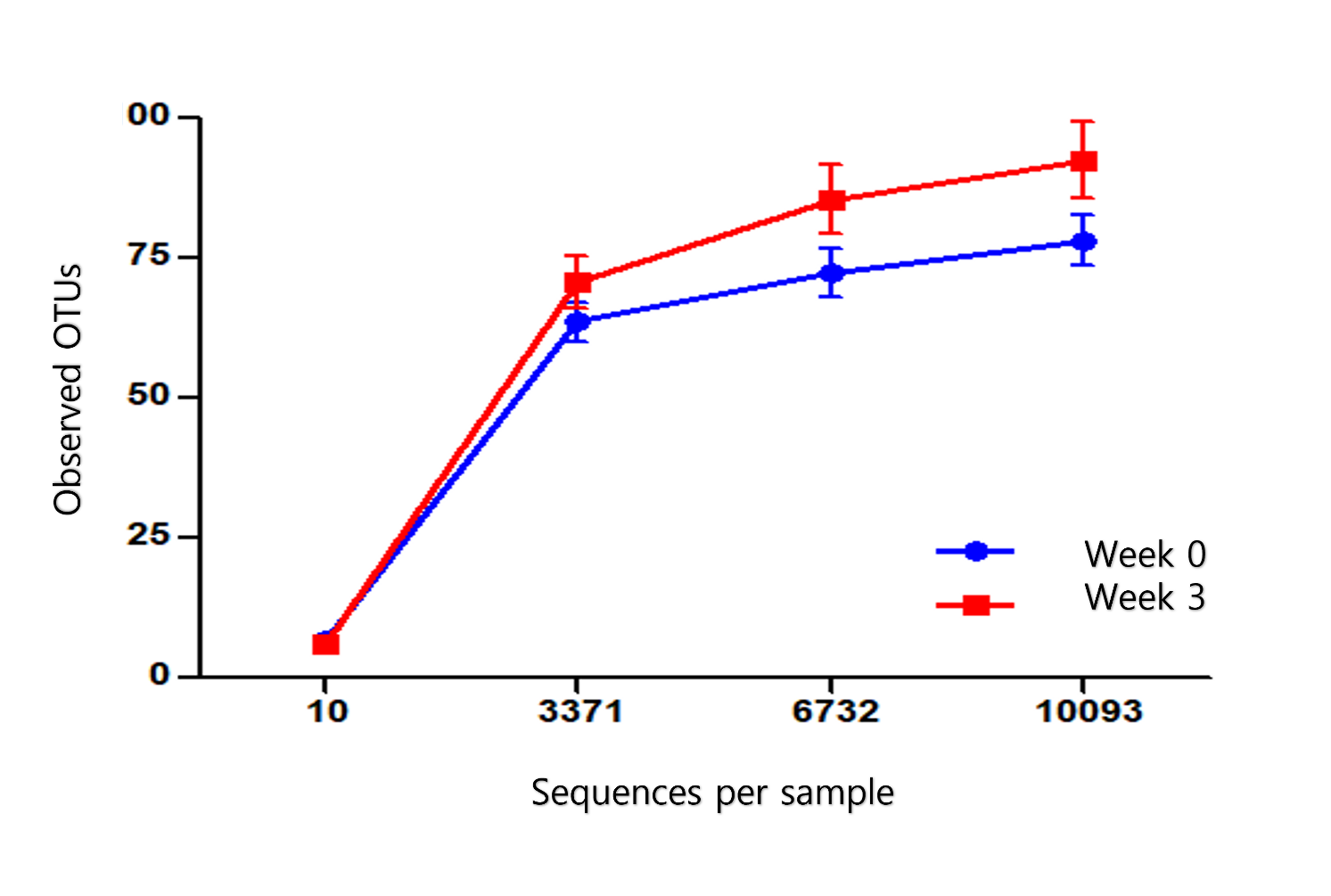


**Supplementary Figure 1.** Microbial richness based on observed OTUs.


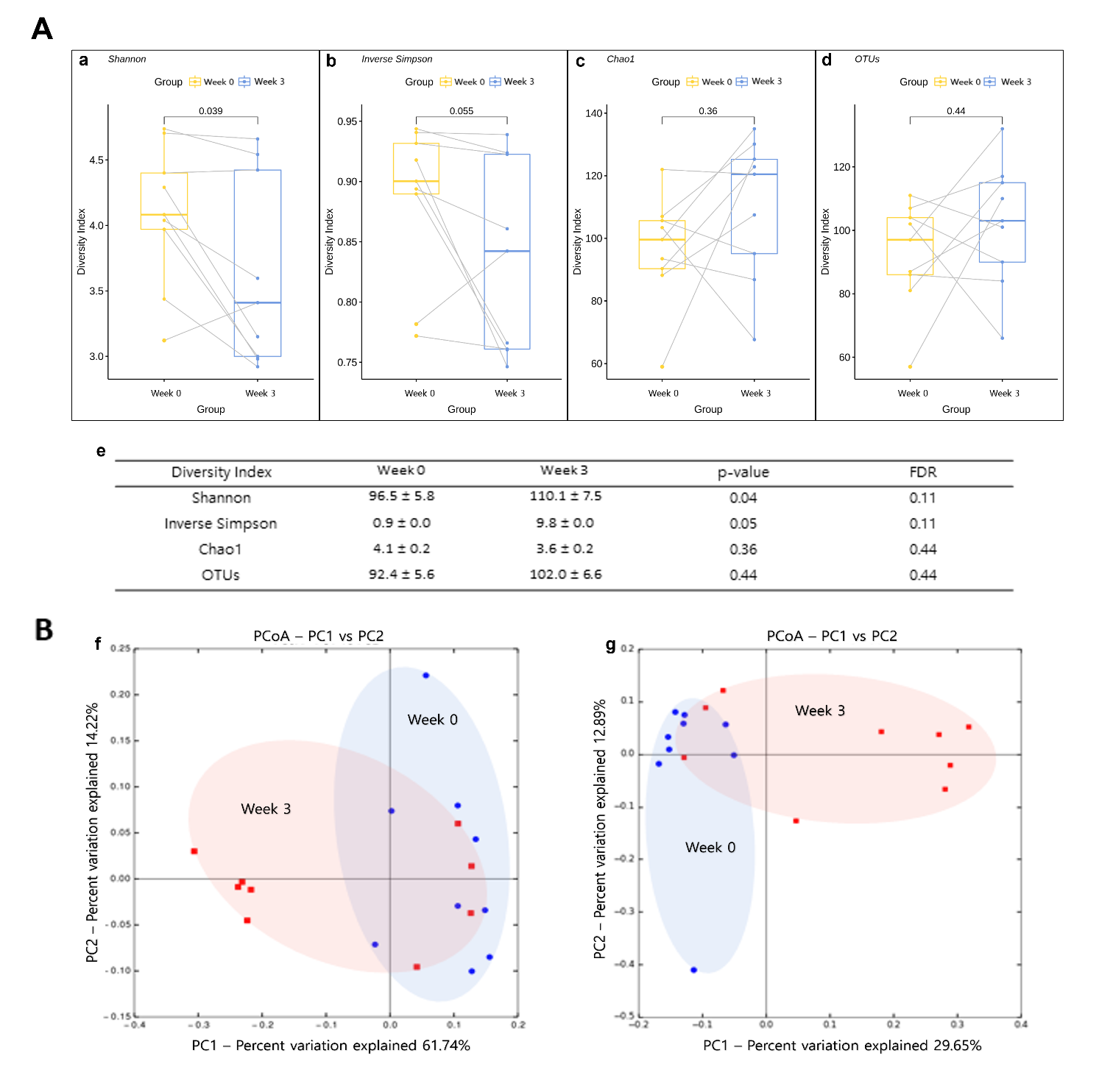


**Supplementary Figure 2.** Alpha and beta diversity between weeks 0 and 3. (**A**) Species richness estimators included are (**a**) Shannon’s diversity index, (**b**) inverse Simpson, (**c**) Chao, and (**d**) OTUs. Statistical analysis was performed using Wilcoxon test by R (version 3.6.2), and *p* value and False Discovery Rate (FDR) are shown in (**e**). (**B**) Principal Coordinate Analysis (PCoA) results based on (**f**) weighted and (**g**) unweighted UniFrac Distances.
